# Neuronal Death and Biomolecular Condensates: Are There Any New Treatment Options for Alzheimer’s Disease?

**DOI:** 10.3390/cells14171356

**Published:** 2025-08-30

**Authors:** Urszula Kochman, Hanna Sitka, Julia Kuźniar, Magdalena Czaja, Patrycja Kozubek, Jan Aleksander Beszłej, Jerzy Leszek

**Affiliations:** 1 Student Scientific Group of Psychiatry, Faculty of Medicine, Wroclaw Medical University, 50-367 Wroclaw, Poland; sitka.hanna@gmail.com (H.S.); julia.kuzniar@gmail.com (J.K.); magdalena.czaja47@gmail.com (M.C.); patrycjakozubek99@gmail.com (P.K.); 2 Department of Psychiatry, Faculty of Medicine, Wroclaw Medical University, 50-367 Wroclaw, Poland; jan.beszlej@umw.edu.pl (J.A.B.); jerzy.leszek@umw.edu.pl (J.L.)

**Keywords:** Alzheimer’s disease, neuronal death, biomolecular condensates, liquid–liquid phase separation

## Abstract

Alzheimer’s disease (AD) is marked by the pathological aggregation of amyloid β (Aβ) and tau proteins. Emerging research reveals that these proteins undergo liquid–liquid phase separation (LLPS), forming biomolecular condensates that promote aggregation and neurotoxicity. These phase-separated structures reshape the intracellular environment, facilitating protein misfolding and spreading. This review highlights recent advances in understanding the role of condensates in AD pathogenesis and explores novel therapeutic strategies targeting condensate dynamics. Promising approaches include small molecules that disrupt LLPS, epigenetic drugs influencing nuclear condensates, and compounds like DDL 920 and RI AG03 that modulate tau phase separation and neuroinflammation, respectively. Additionally, anti-inflammatory agents, such as nucleotide reverse transcriptase inhibitors (NRTIs), offer potential for upstream intervention. Targeting biomolecular condensates presents a next-generation strategy for AD treatment. Future research should focus on in vivo profiling of condensate composition, biomarker development, and the development of patient-specific therapies to enable early, disease-modifying interventions.

## 1. Introduction

Alzheimer’s disease (AD) is an increasingly significant problem in contemporary medicine. It directly influences patients’ daily lives by impairing their memory, cognitive abilities, decision-making, and mood, which is often complicated by multimorbidity [1,2]. AD is the most common type of neurodegenerative disease (60–70% of all dementias [2]), affecting over 50 million people worldwide [3]. The aetiology and pathogenesis of AD have not yet been fully ascertained. A principal factor of the pathophysiology of AD is the accumulation of extracellular senile plaques and intracellular neurofibrillary tangles in the central nervous system, which leads to synaptic loss, neuronal cell death, and ultimately severe cognitive impairment [4]. Amyloid-beta (Aβ) is a key contributor to the formation of pathological deposits [5]. There are already various specifically targeted therapeutic approaches being developed. Nonetheless, disease-modifying pharmacological treatment is currently insufficient, and causal treatment of AD is not entirely possible at this stage. Despite medical advances, AD remains incurable. Current treatment strategies aim only to alleviate symptoms, but they do not address the underlying cause. Recently, monoclonal antibodies, such as lecanemab or donanemab, have been developed. They include pharmacological approaches, such as acetylcholinesterase inhibitors (donepezil, galantamine, rivastigmine) and memantine, as well as non-pharmacological interventions to support cognitive reserve [6,7]. In ageing populations, AD is becoming an issue that requires an increasing amount of healthcare resources; care of one AD patient requires a complex approach involving many individuals, such as primary, specialist and community-based rehabilitants. In many cases, palliative care also needs to be involved [2].

It is essential to distinguish between biomolecular condensates and protein aggregates, since these terms are not interchangeable. Biomolecular condensates are dynamic, reversible, membraneless compartments formed through processes such as liquid–liquid phase separation (LLPS). They form part of normal physiology, and the constituent proteins usually retain their native conformation. These condensates facilitate processes such as RNA metabolism, signalling, the stress response and gene expression, and their reversibility is a key feature [8]. In contrast, protein aggregates are often pathological endpoints. They arise from misfolding and denaturation, resulting in the formation of insoluble deposits that are often irreversible and difficult for the cell to clear. These deposits are a hallmark of neurodegenerative diseases such as Alzheimer’s and Parkinson’s diseases, as well as amyloidoses [9]. Although condensates and aggregates both involve higher-order assemblies, they differ significantly in terms of reversibility and functional role. While some protein aggregates may be functional and reversible in rare cases—for instance, specific yeast amyloid-like structures involved in stress adaptation—these represent regulated exceptions, not the norm [9]. Furthermore, it is now recognised that biomolecular condensates can influence the aggregation process. For instance, they can increase the local concentration of molecules, promote interfacial nucleation or alter energy landscapes. This modulates the kinetics of aggregate formation in the context of neurodegenerative disease [10].

Over time, an increasing number of studies have been conducted, revealing new aspects of the pathophysiology of AD. One of the latest theories suggests that biomolecular condensates play a crucial role in AD pathogenesis, as they contribute to the formation of protein aggregates. Protein aggregates responsible for the pathogenesis of many degenerative disorders have been known for over a hundred years. However, the exact pathways of their formation and the determinants of these processes are still not fully understood [11]. Biomolecular condensates are cellular structures formed through liquid–liquid phase separation, which involves various biomolecules, including proteins, nucleic acids, small molecules, and lipids [12]. They are believed to play a key role in the spatial organisation of the cellular interior; they function as storage compartments, organisational centres, and reaction sites by locally concentrating or excluding specific biomolecules, modulating transition state energies, and stabilising their concentrations [13,14].

Due to the growing number of studies on molecular condensates and the new insights they provide, it is becoming possible to gain a more precise understanding of the pathophysiology of AD and to discover new, more targeted therapeutic options. This study aims to investigate the role of condensates in neuronal death and AD pathogenesis and, consequently, therapeutic possibilities related to them.

## 2. Neuronal Death in Alzheimer’s Disease

### 2.1. Molecular and Cellular Factors Leading to Neuronal Death in Alzheimer’s Disease

AD is a gradually worsening neurodegenerative condition, characterised by several key pathological changes, such as the buildup of amyloid plaques and neurofibrillary tangles, increased oxidative stress, inflammation in the nervous system, and a significant reduction in cholinergic signalling.

#### 2.1.1. Accumulation of Amyloid and Tau Proteins

One of the most extensively investigated hypotheses in the field is the amyloid-beta (Aβ) protein hypothesis [15]. Under physiological conditions, the Aβ precursor protein can undergo processing via two separate enzymatic pathways. In the non-amyloidogenic route, the Aβ precursor protein is first cleaved by α-secretase and then by γ-secretase, resulting in the generation of non-toxic P3 fragments. In contrast, the amyloidogenic pathway begins with cleavage by β-secretase, followed by γ-secretase, resulting in the production of Aβ peptides, which are associated with plaque formation in AD [16]. There are several isoforms of Aβ peptides, including Aβ43, Aβ40, and Aβ42; their formation depends on the precise site at which γ-secretase cleaves the Aβ precursor protein [16,17]. Aβ42 is considered to be a key contributor to the progression of the disease, as amyloid plaques composed of it exhibit significant neurotoxicity. However, the Aβ40 isoform is the most prevalent, accounting for up to 90% of total Aβ, and, together with Aβ42, it contributes to disease progression [18].

Another protein aggregation-related theory involves the tau protein. Tau protein is a microtubule-associated protein that is believed to play a key role in regulating microtubule structure and dynamics [19]. The tau protein hypothesis suggests that pathological alterations in tau contribute to neurodegenerative diseases [20], in which the ratio of 4R and 3R tau isoforms is imbalanced, unlike under physiological conditions. This imbalance leads to increased tau phosphorylation and the formation of insoluble aggregates, such as neurofibrillary tangles (NFTs) and paired helical filaments (PHFs) [21,22].

Clinical studies have shown a direct correlation between tau tangles and the severity of cognitive impairment [23,24]. Tau pathology is associated with neuroinflammation [25] and develops more rapidly compared to Aβ aggregation [26].

#### 2.1.2. Oxidative Stress

Another contributor responsible for cell death in AD is oxidative stress. It is not only an initiating factor, but it also exacerbates neurodegenerative processes. Multiple studies have shown that the brains of AD patients exhibit the effects of oxidative stress, which is manifested by elevated levels of oxidised proteins, products of lipid peroxidation, and DNA and RNA damage [27,28,29]. The term oxidative stress refers to the imbalance in production of reactive oxygen species (ROS) and antioxidant defences of brain cells, which results in damage to fundamental cellular components, including lipids, proteins and nucleic acids [29,30]. ROS are formed as a result of mitochondrial damage and abnormal accumulation of transition metals such as iron, copper and zinc, whose high concentration has been detected in the brains of AD patients. These metals, as well as mercury and aluminium, catalyse chemical reactions which generate free radicals, additionally aggravating oxidative stress [27,29].

Additionally, Aβ plaques and neurofibrillary tau tangles may intensify oxidative stress, and at the same time, oxidative stress augments the production of Aβ and tau aggregates, creating a pathogenic vicious cycle [31]. In multiple transgenic models of AD harbouring APP and PS-1 mutations, age-related accumulation of Aβ has been consistently linked to increased levels of hydrogen peroxide, nitric oxide and heightened oxidative damage to proteins and lipids—supporting the role of Aβ in driving oxidative stress [32]. The human brain is especially vulnerable to oxidative stress because of the high content of easily oxidizable lipids, intensive oxygen metabolism and limited capacity for regeneration and antioxidant defence [29,30]. With age, levels of crucial antioxidants, such as glutathione, decline [27]. Redox imbalance is observed already in early stages of AD, and even in mild cognitive impairment [27,33].

### 2.2. The Impact of Epigenetics on Neuronal Survival

Epigenetic mechanisms affect the survival of neurons in AD, as they influence the expression of genes responsible for synaptic plasticity, neurogenesis and neuroinflammation [34]. The brains of AD patients exhibit neuronal changes, specifically in the methylation of DNA enhancer regions, particularly within genes involved in Aβ biosynthesis, such as BACE1, which promotes the accumulation of pathological deposits and contributes to the progression of neurodegeneration [35,36,37]. Moreover, epigenetic deregulation may lead to reactivation of the cell cycle in mature neurons, initiating apoptosis and exacerbating neuronal loss [37].

### 2.3. Microglia and Neuroinflammation in Disease Progression

Microglia, the primary population of immune cells in the central nervous system, play a crucial role in AD pathogenesis by regulating neuroinflammatory processes. In response to Aβ aggregation and neuronal damage, microglia become activated, which leads to the secretion of pro-inflammatory cytokines, chemokines and ROS. This exacerbates chronic brain inflammation [25]. Long-lasting microglial activation, rather than protecting neurons, may lead to their impairment by fostering a neurotoxic environment and disrupting synaptic homeostasis [38]. To date, research has shown that microglial functioning changes and chronic neuroinflammation are closely linked to AD progression, and modulation of the microglial response may be a promising therapeutic target for delaying neurodegeneration [25,38].

## 3. Biomolecular Condensates and Protein Aggregation

### 3.1. Definition and Characteristics of Biomolecular Condensates

Biomolecular condensates are dynamic, membraneless cellular compartments that organise specific proteins, nucleic acids, and other biomolecules into concentrated assemblies without membrane boundaries. These condensates facilitate compartmentalisation and regulation of biochemical reactions by creating distinct microenvironments within the cell. Their liquid-like properties allow rapid assembly and disassembly, enabling cells to respond flexibly to environmental and physiological cues [39,40,41].

### 3.2. Role of Condensates in Protein Aggregation, Including Aβ42 and Tau, and Their Impact on Alzheimer’s Disease Pathogenesis

Biomolecular condensates have been implicated in the aggregation of neurodegenerative disease-associated proteins such as Aβ42 and tau. These proteins can undergo phase separation, forming concentrated droplets that act as nucleation hubs, accelerating the formation of toxic aggregates like amyloid plaques and neurofibrillary tangles, key pathological markers of AD. Aberrant phase transitions of these condensates—from fluid-like to more solid or gel-like states—may contribute to the persistence and toxicity of pathological aggregates [42,43,44,45].

### 3.3. Mechanisms Through Which Condensates Both Promote and Inhibit Protein Aggregate Formation

Biomolecular condensates exhibit a dual role in protein aggregation. On one hand, the high local concentration of aggregation-prone proteins within condensates facilitates nucleation. It accelerates fibril formation, especially under pathological conditions where the liquid phase can mature into more solid, amyloid-like structures. This mechanism promotes the progression of neurodegenerative diseases [46,47]. On the other hand, condensates can act as protective compartments by sequestering misfolded or aggregation-prone proteins, thereby limiting their interactions with other cellular components and potentially preventing toxic aggregation. Furthermore, molecular chaperones and proteostasis factors enriched within condensates can promote the refolding or degradation of aberrant proteins, thus counteracting aggregate formation. The delicate balance between these opposing functions is critical for maintaining cellular homeostasis and preventing disease [40].

### 3.4. Molecular Stress Makes Old Neurons Vulnerable to Neurodegenerative Diseases

Ageing significantly impairs neuronal resilience, rendering neurons more susceptible to stress and neurodegeneration. A landmark 2025 study by Rhine et al. demonstrates that age-related mislocalization of splicing proteins and persistent, chronic cellular stress represent a fundamental mechanism by which ageing predisposes neurons to disease-related decline [48].

### 3.5. Splicing Factor Mislocalization and RNA Dysregulation

Using aged human neurons derived from transdifferentiated fibroblasts, Rhine et al. reveal widespread nuclear depletion of key RNA-binding proteins, particularly spliceosomal components such as TDP-43. These proteins abnormally relocate to the cytoplasm, where they accumulate instead of being sequestered into stress granules—unlike in young neurons—leading to broad alternative splicing defects that disrupt neuronal function [48,49,50]. These molecular alterations are validated in aged human and mouse brain tissue, marking a significant shift in RNA homeostasis with age.

### 3.6. Chronic Stress and Impaired Stress Response

This study further highlights chronic, unchecked cellular stress in aged neurons, characterised by persistent phosphorylation of stress kinases and the accumulation of untranslated RNAs and proteins. This “always-on” stress state exhausts the neurons’ capacity to respond to new insults—analogous to a body constantly fighting illness and becoming vulnerable to additional infections [51,52]. HSP90α dysfunction, compromised ubiquitination, and failure of stress granule dynamics are implicated in this decline, preventing proper sequestration of splicing proteins and promoting neuronal fragility [48,53].

### 3.7. Mechanistic Contribution to Neurodegeneration

These findings link molecular stress and RNA dysregulation in aged neurons to the aetiology of neurodegenerative diseases. Mislocalized splicing factors, such as TDP-43, are well-known contributors to frontotemporal dementia and ALS, and their age-induced redistribution may prime neurons for similar pathologies [48]. Chronic stress and impaired RNA processing degrade proteostasis, synaptic integrity, and survival pathways—hallmarks of early AD.

### 3.8. Broader Context and Vulnerability Models

This work aligns with prior evidence that neuronal selective vulnerability increases with oxidative stress, mitochondrial decline, and DNA damage inherent in ageing. The failure to mount an appropriate stress response, rooted in splicing and chaperone deficiencies, adds a critical layer to the connection between ageing and neurodegeneration.

### 3.9. Therapeutic Implications

#### 3.9.1. Splicing Protein Stabilisation

Targeting nuclear pore complexes (NPCs) or nuclear retention signals presents a promising strategy for preventing the mislocalization of spliceosomal proteins, particularly TDP-43. Restoration of NPC integrity and modulation of retention mechanisms have demonstrated rescue of nuclear localisation in aged or stressed neurons, reducing cytoplasmic accumulation and stress marker induction [48]. Early small-molecule screens have identified compounds that partially restore nuclear RNA-binding proteins in disease models, suggesting potential utility in ageing-related splicing defects [54].

#### 3.9.2. Stress-Response Enhancement

Enhancing HSP90α activity and proteasome/ubiquitin pathway function can improve stress granule dynamics and preserve splicing fidelity. Chronic activation of HSP90-mediated chaperone networks promotes proper protein folding and stress adaptation, while selective modulation of HSP90α has been shown to activate HSF1 and enhance proteostasis in neurodegenerative models [55,56]. Furthermore, HSP90 regulates stress-granule disassembly via client kinases (like DYRK3), helping maintain RNA splicing regulation under chronic stress [57].

#### 3.9.3. RNA-Targeted Interventions

Antisense oligonucleotides (ASOs) and RNA-binding small molecules can directly correct splicing defects and mitigate downstream toxicity. Nusinersen, a seminal SMN2-targeting ASO, demonstrates efficient CNS delivery and durable splice modulation for spinal muscular atrophy, setting a precedent for applications in age-related RNA dysregulation [48,58]. Concurrently, small molecules that modulate splicing-factor kinases (e.g., SRPK, CLK) and stabilise RNA structures have shown efficacy in reversing aberrant splicing in preclinical neurodegenerative models [59].

## 4. Therapeutic Implications: Targeting Biomolecular Condensates

The growing recognition of biomolecular condensates as pivotal microenvironments in the early pathogenesis of AD offers a promising new frontier for therapeutic intervention. These condensates, formed through LLPS, have been shown to nucleate amyloid formation, alter protein stability, and modulate aggregation kinetics, particularly for Aβ and tau proteins. Targeting these dynamic compartments represents a novel approach to halting or reversing protein misfolding and neurotoxicity.

### 4.1. Modulating Phase Separation with Small Molecules: The Case of Melatonin

LLPS of amyloidogenic proteins, such as Aβ42, can be modulated by small molecules, potentially preventing the progression to pathogenic aggregates. Melatonin, a neuroprotective molecule with antioxidant and anti-inflammatory properties, has shown promise in influencing condensate dynamics in several in vitro and in vivo models. While melatonin was not directly tested in the recent Aβ-condensate studies, its known modulatory role in protein aggregation pathways makes it a promising candidate for future exploration [36].

Recent experiments have shown that Aβ42 undergoes lipid-driven phase separation, forming spherical condensates that act as nucleation sites for amyloid fibrils [3]. Compounds such as 1,6-hexanediol, which disrupt hydrophobic interactions within condensates, have been demonstrated to dissolve Aβ42 condensates both in vitro and in neuronal cells, indicating a feasible pharmacological approach to mitigating early-stage aggregation [3].

### 4.2. Altering Condensate Composition and Properties to Prevent Aggregation

Condensates provide a distinct microenvironment where local concentration, viscosity, and interfacial properties strongly influence protein behaviour. Phase-separated Aβ42 condensates, rich in phospholipids, demonstrate a liquid-to-solid transition that accelerates fibril formation [3]. Therapeutic strategies aimed at modifying condensate composition, such as altering ionic strength, introducing competitive peptides, or manipulating lipid content, may inhibit their pathological maturation.

The “host-guest” behaviour of proteins in condensates can also be therapeutically exploited. For instance, co-condensation of tau with inert mutants in dense phases has been shown to reduce the effective concentration of aggregation-prone forms, delaying fibrillization [12]. This strategy mimics buffering mechanisms that cells might use to prevent aggregation under physiological conditions.

### 4.3. Epigenetic Modulation: HDAC Inhibitors and Chromatin-Linked Condensates

Epigenetic modifiers, particularly histone deacetylase (HDAC) inhibitors, have been shown to influence condensate dynamics indirectly by altering chromatin structure and gene expression patterns. Increasing attention has been directed toward the role of specific HDAC isoforms in the pathology of tau [36]. HDAC6, a predominantly cytoplasmic deacetylase, regulates the acetylation of non-histone proteins, including tau. Its inhibition has been associated with elevated tau acetylation, which may reduce tau aggregation and facilitate its clearance via the autophagy–lysosome system. In parallel, HDAC2, which is primarily nuclear, has been implicated in the repression of genes involved in synaptic plasticity and memory. Inhibition of HDAC2 not only enhances cognitive performance in animal models but also appears to mitigate tau-related neurotoxicity. Together, these findings suggest that selective targeting of HDAC6 and HDAC2 may represent a promising strategy for modulating tau pathology, potentially through regulation of tau acetylation status, nuclear condensate organisation, and broader proteostasis networks [60,61,62]. Other epigenetic interventions, including BET inhibitors and DNA methylation modulators, are being explored for their impact on LLPS-prone proteins and their compartmentalisation. These agents could modify the nuclear environment in ways that suppress aberrant phase transitions.

### 4.4. Overview of Current Preclinical and Clinical Research

Experimental studies have confirmed that Aβ42 forms condensates on lipid membranes and neuronal surfaces, significantly accelerating amyloid nucleation [3]. These structures are liquid-like, sensitive to hexanediol, and represent early intermediates in the aggregation cascade. Their characterisation has been aided by confocal microscopy, fluorescence recovery after photobleaching (FRAP), and total internal reflection fluorescence (TIRF) imaging.

Research on the role of condensates in tau and TDP-43 aggregation has further highlighted the five key physical-chemical factors that influence aggregation within condensates: increased local concentration, altered diffusivity, interfacial effects, microenvironmental changes in energy landscapes, and chaperone co-localisation [12]. These features provide multiple potential targets for therapeutic design.

While no therapies yet approved directly target biomolecular condensates, especially in the advanced stage of the disease, multiple candidates are under preclinical investigation, including LLPS-modulating peptides, lipid-targeted agents, and epigenetic drugs. These strategies, shown in Table 1, aim to intervene at the earliest stages of aggregation, offering hope for disease-modifying treatments in AD.

## 5. Novel Approaches to Treating Alzheimer’s Disease

Recent years have witnessed a surge in novel therapeutic strategies, currently known, targeting key pathological processes in AD. These include modulation of neuroinflammation, amyloid clearance, and neuroprotection via phytochemicals and innovative small molecules.

### 5.1. NRTIs and Their Impact on Inflammasomes

Nucleoside reverse transcriptase inhibitors (NRTIs), initially developed for the treatment of HIV and hepatitis B, have recently emerged as potential modulators of neuroinflammation. A large-scale retrospective cohort study utilising the U.S. Veterans Health Administration (VHA) and IBM MarketScan databases demonstrated a significant association between chronic NRTI exposure and reduced incidence of AD. After adjusting for demographic and clinical covariates, NRTI use was associated with a 9.3% annual reduction in AD risk in the VHA cohort (adjusted HR = 0.907; 95% CI: 0.889–0.925; *p* < 0.001) and a 12.3% annual reduction in the MarketScan cohort (adjusted HR = 0.877; 95% CI: 0.831–0.925; *p* < 0.001) [65,66].

Mechanistically, NRTIs have been shown to inhibit the activation of the NLRP3 inflammasome in microglia and other innate immune cells, thereby preventing inflammasome assembly, caspase-1 activation, and the subsequent release of pro-inflammatory cytokines, including IL-1β and IL-18. This inhibition leads to attenuation of Aβ- and tau-mediated neurotoxicity in preclinical models. A next-generation NRTI derivative, K-9, retains the inflammasome-inhibitory properties of classical NRTIs but with reduced off-target effects and improved safety in vivo. K-9 is currently undergoing preclinical development as a candidate for disease-modifying therapy in AD [65,67].

### 5.2. Remternetug: Targeting Amyloid Deposition

Remternetug (LY3372993) is an investigational IgG1 monoclonal antibody directed against pyroglutamate-modified Aβ plaques. In a Phase 1 multiple ascending-dose trial, 75% of treated early AD patients showed ≥24.1 centiloid plaque reduction after 169 days, with good safety and no anti-drug antibodies. Currently, the TRAILRUNNER-ALZ programme includes Phase 3 studies assessing subcutaneous administration in early-stage and pre-symptomatic carriers, focusing on plaque removal and cognitive decline [68,69,70].

### 5.3. Plant-Derived Compounds: Carnosic Acid

Carnosic acid from rosemary and sage exhibits neuroprotective, anti-inflammatory, and Nrf2-mediated antioxidative effects. In cellular and rodent AD models, it downregulates pro-inflammatory cytokines and inhibits NLRP3 inflammasome activation. It also enhances autophagy and mitochondrial function. While promising mechanistic results exist, formal safety and efficacy assessments in humans remain limited [71,72].

### 5.4. New Small Molecules: RI AG03 and DDL 920

Two novel small molecules are emerging as potential AD therapies:

- RI AG03 selectively inhibits NLRP3 inflammasome assembly and subsequent IL-1β release, reducing microglial activation, amyloid β deposition, and synaptic dysfunction in mouse and fruit-fly models of tauopathy [73]. For example, in Drosophila engineered to overexpress human tau, RI AG03 suppressed neuronal degeneration and extended lifespan by targeting dual tau aggregation hotspots [73].
- DDL 920 disrupts aberrant tau aggregation by interfering with tau–protein LLPS. It reduces tau oligomer formation and neurofibrillary tangle load and improves cognition in mouse tauopathy models. Pharmacokinetic studies report good brain penetration, metabolic stability, and no observable toxicity [74].

Both compounds are currently in preclinical development, with ongoing optimisation for safety, efficacy, and pharmacodynamics ahead of first-in-human trials. Their comparison is presented in Table 2.

### 5.5. Peptide-Based Condensate “Killswitch”

A novel therapeutic approach involves the use of synthetic peptides that target biomolecular condensates. Zhang et al. [75] (2025) recently described a 17-amino-acid peptide-based ‘killswitch’ that can selectively modulate condensate dynamics in the journal Nature [75]. This micropeptide, which is enriched in hydrophobic and aromatic residues, self-associates in a manner that is not homologous to human proteins and markedly reduces protein mobility within condensates. Using a nanobody-guided recruitment system, the peptide was directed to various endogenous and pathological condensates, including nucleoli, nuclear speckles, chromocentres, oncogenic fusion protein condensates and adenoviral nuclear bodies. There, it altered condensate composition, arrested internal protein dynamics and disrupted disease-related processes, such as the proliferation of condensation-driven leukaemia cells and viral particle assembly [75].

Although this approach is still in its infancy, it demonstrates the therapeutic potential of manipulating condensate microenvironments directly rather than targeting individual molecular components. In the future, such a strategy may be adapted to neurodegenerative disorders, including AD, where aberrant phase separation and condensate dysfunction are increasingly recognised as central pathological mechanisms.

### 5.6. Safety and Efficacy Evaluation

- NRTIs: Epidemiological analyses suggest long-term safety in HIV patients, yet AD-specific clinical trials remain necessary to assess cognitive benefit and potential off-target effects [65,67,68].
- Remternetug: Early-phase safety results look promising; forthcoming Phase 3 trials aim to evaluate plaque clearance effectiveness and cognitive outcomes and monitor for amyloid-related imaging abnormalities (ARIA) [68,69,70].
- Carnosic acid: Extensive preclinical studies support its antioxidant and anti-inflammatory activity with favourable safety. Human clinical trials are needed to determine optimal dosing and efficacy [71,72].
- RI AG03 and DDL 920: These molecules have demonstrated preclinical efficacy and tolerability. Ongoing toxicology and pharmacodynamic assessments are being performed to prepare for regulatory submission and first-in-human studies [73,74].

## 6. Clinical Trials

We focused on recent, significant clinical trials in AD to enhance translational relevance.

The Phase III Clarity AD randomised, placebo-controlled trial evaluated lecanemab, a humanised IgG1 monoclonal antibody that targets Aβ protofibrils, in individuals with mild cognitive impairment or early dementia due to AD. Over 18 months, lecanemab slowed clinical decline by 27% (difference in CDR-SB change from baseline: −0.45; 95% CI: −0.67 to −0.23; *p* < 0.001) and significantly reduced amyloid burden by 59 centiloids, as well as producing significant reductions in ADAS-Cog14, ADCOMS and ADCS-MCI-ADL scores [76,77]. Adverse events included infusion reactions (approximately 26%), ARIA-E (approximately 12.5%) and ARIA-H (approximately 17%), compared to approximately 1.7% and 9% for placebo, respectively [78,79,80]. The FDA granted accelerated approval in January 2023, followed by traditional approval later in the year [81].

Another notable study is the TRAILBLAZER-ALZ 2 trial, a randomised and placebo-controlled investigation of donanemab (LY3002813) in patients with early AD. This monoclonal antibody has been shown to reduce amyloid levels and slow disease progression. However, it was also associated with ARIA and infusion reactions, as detailed in the New England Journal of Medicine and confirmed during the approval review process [82].

Finally, the Phase II Laureate trial of semorinemab, an anti-tau monoclonal antibody, in patients with mild to moderate AD showed a modest improvement in the ADAS-Cog11 score, particularly in memory-related domains. However, there was no significant improvement in functional or global outcomes (e.g., ADCS-ADL, CDR-SB, MMSE). The treatment did lower CSF tau markers, suggesting central engagement but limited clinical efficacy [83].

Overall, these clinical findings emphasise that, although anti-amyloid and anti-tau therapies can modestly alter disease progression, their efficacy is limited, and there are significant safety concerns, particularly regarding ARIA. This emphasises the need for additional approaches, such as strategies that modulate condensates, which could provide new ways to address multiple pathological pathways in AD. A summary of selected recent clinical trials is presented in Table 3.

## 7. Discussion

The concept of biomolecular condensates has profoundly reshaped our understanding of AD pathogenesis. These dynamic, membrane-less compartments—formed through LLPS—not only organise intracellular biochemistry but also act as pathological hubs for aggregation-prone proteins such as Aβ and tau.

Recent research has demonstrated that Aβ42 can form lipid-associated condensates on neuronal membranes, serving as nucleation sites that accelerate fibrillization. These condensates often undergo a liquid-to-solid phase transition, culminating in the formation of neurotoxic aggregates [12,84]. Similarly, tau and other intrinsically disordered proteins exhibit LLPS behaviour, with their phase separation facilitating conformational misfolding and intercellular propagation [12,43,85]. Significantly, the unique microenvironments within condensates—characterised by altered viscosity, macromolecular crowding, ionic conditions, and chaperone exclusion—significantly reshape protein folding landscapes, enhancing the propensity for pathological aggregation [12,43].

Targeting biomolecular condensates is now emerging as a next-generation strategy in AD therapeutics. Small molecules such as 1,6-hexanediol and melatonin analogues have been shown to disrupt LLPS or prevent the maturation of condensates, thereby hindering the formation of toxic aggregates [3,36,86]. Epigenetic drugs, including HDAC inhibitors such as Tubastatin A and Ricolinostat, influence nuclear condensates and suppress tau expression, offering neuroprotective benefits in preclinical models [36,65].

Novel small molecules, such as DDL 920, further exemplify this therapeutic direction. DDL 920 interferes with pathological tau phase separation, reduces tau oligomer formation, and ameliorates cognitive deficits in tauopathy models [4]. Similarly, RI AG03, a selective NLRP3 inflammasome inhibitor, reduces microglial activation, Aβ deposition, and synaptic loss in AD mouse models [87,88].

Moreover, anti-inflammatory agents such as NRTIs have demonstrated promising effects by modulating innate immune responses linked to condensate-driven neurotoxicity [65]. These approaches align with early upstream events in AD pathogenesis and may offer interventions with fewer adverse effects compared to late-stage amyloid- or tau-targeted strategies. In this context, it is essential to establish a connection between condensate formation and gene regulatory networks that facilitate the proper development of immune cells.

Looking ahead, several key areas demand further investigation to translate these insights into viable treatments. Molecular mapping of condensate composition across different stages of AD and brain regions will provide crucial information. Dissecting LLPS dynamics in vivo, especially regarding neuron subtype vulnerability and stress response, will illuminate the mechanisms of selective neurodegeneration. Additionally, high-throughput screening of LLPS-modulating compounds in physiologically relevant models, such as iPSC-derived neurons or brain organoids, is crucial for therapeutic discovery. Biomarker development for tracking condensate dynamics in CSF or via neuroimaging could greatly enhance clinical translation.

Recent advances in phase separation biology suggest that biomolecular condensates may leave distinct molecular signatures detectable in cerebrospinal fluid (CSF). Proteins involved in condensate formation, such as phosphorylated tau (pTau), TDP-43, FUS, or stress granule components (e.g., G3BP1), may be released into CSF due to neuronal stress, degeneration, or leakage from disrupted condensates [48,89]. Additionally, altered ratios of condensate-associated isoforms (e.g., 3R/4R tau), phase transition markers (e.g., ubiquitinated condensate proteins), or RNA-binding proteins mislocalized from the nucleus could serve as surrogate indicators of LLPS dysregulation [90]. High-throughput proteomic and RNA-seq analyses of CSF hold promise for identifying condensate-derived biomarkers, offering novel diagnostic and prognostic tools that reflect early, upstream changes in AD pathology [66,91].

Finally, precision medicine approaches—tailoring interventions based on genetic risk factors like APOE4—will be key to maximising the efficacy of condensate-targeted therapies.

As our mechanistic understanding of biomolecular condensates expands, so too does the potential for developing precise, disease-modifying therapies that intervene early in the progression of AD. According to all authors, more research is needed to better understand the effectiveness of treatment in preventing and treating AD.

## Figures and Tables

**Table 1:** Therapeutic strategies targeting biomolecular condensates in Alzheimer’s disease.

Strategy	Mechanism of Action	Target Molecules/Processes	Supporting Evidence	Reference
Small molecule modulation (e.g., melatonin)	Modulates LLPS; stabilises monomeric forms and reduces pathological condensation	Aβ42, tau	Neuroprotective potential; indirect support from LLPS-related studies	[63]
Condensate disruption (e.g., 1,6-hexanediol)	Disrupts hydrophobic interactions to dissolve condensates	Aβ42-lipid condensates	In vitro and cellular dissolution of Aβ42 condensates	[48]
Condensate composition alteration (e.g., tau mutant co-condensation)	Dilutes aggregation-prone proteins within condensates by inert co-localising species	Tau, Aβ42	Delay of fibril formation in dense phase via co-condensation	[64]
Epigenetic modulation (e.g., HDAC inhibitors)	Alters chromatin condensates and transcriptional states; may reduce phase separation of aggregation-prone proteins	Tau, chromatin proteins	Reduced tau aggregation, improved cognitive outcomes in AD models	[63]
Chaperone localization to condensates	Enhances local folding capacity, counteracts aggregation inside condensates	TDP-43, tau, Aβ42	Conceptual and experimental support from host–guest systems	[50]
Interface-targeted aggregation inhibition	Prevents nucleation at condensate interfaces by altering interfacial chemistry	Aβ42, hnRNPA1, tau	Interfaces shown to enhance aggregation; strategy under investigation	[50]

**Table 2:** Emerging small molecules targeting biomolecular condensates in Alzheimer’s Disease.

Compound	Mechanism of Action	Effects in Preclinical Models	Pharmacokinetics	Development Status
RI AG03	Selectively inhibits NLRP3 inflammasome assembly and IL-1β release	Reduces microglial activation, amyloid β deposition, and synaptic dysfunction in mouse and Drosophila tauopathy models; suppresses neuronal degeneration and extends lifespan	Ongoing pharmacodynamic profiling; preclinical safety encouraging	Preclinical stage; under optimisation for safety, efficacy, and first-in-human trials [71]
DDL 920	Disrupts tau–protein liquid–liquid phase separation	Decreases tau oligomerization and neurofibrillary tangle burden; improves cognitive performance in mouse models of tauopathy	Demonstrates good brain penetration, metabolic stability, and no observable toxicity	Preclinical stage; progressing toward clinical readiness [72]

**Table 3:** Summary of selected recent clinical trials in Alzheimer’s disease.

Trial Name	Drug (Target)	Phase/Population	Key Outcomes	Safety/Limitations
Clarity AD	Lecanemab (anti-Aβ protofibril mAb)	Phase III, early AD (MCI and mild dementia)	27% slowing of decline on CDR-SB; significant amyloid clearance (−59 centiloids); improved ADAS-Cog14, ADCOMS, ADCS-MCI-ADL	ARIA-E (~12.5%), ARIA-H (~17%), infusion reactions (~26%)
TRAILBLAZER-ALZ 2	Donanemab (anti-Aβ plaque mAb)	Phase III, early AD	Reduction in amyloid burden; slowed progression of clinical decline	ARIA (dose-dependent), infusion reactions
Lauriet	Semorinemab (anti-tau mAb)	Phase II, mild to moderate AD	Modest benefit on ADAS-Cog11 (memory domain); reduced CSF tau markers; no significant functional/global benefit	Limited efficacy, no ADCS-ADL or CDR-SB improvement

## Data Availability

Data sharing is not applicable, as no datasets were generated or analyzed during the currentl study.

## Abbreviations

AD	Alzheimer’s Disease
Aβ	amyloid β
LLPS	liquid–liquid phase separation
NRTIs	nucleotide reverse transcriptase inhibitors
NFTs	neurofibrillary tangles
PHFs	paired helical filaments
ROS	reactive oxygen species
NPCs	nuclear pore complexes
ASOs	Antisense oligonucleotides
HDAC	histone deacetylase
FRAP	fluorescence recovery after photobleaching
TIRF	total internal reflection fluorescence
VHA	Veterans Health Administration
